# Laparoscopic removal of worm in biliary canal

**DOI:** 10.4103/0972-9941.62533

**Published:** 2010

**Authors:** Viroj Wiwanitkit

**Affiliations:** Wiwanitkit House, Bangkhae, Bangkok, Thailand 10160

Sir,

With great interest, I read the recent publication on ‘using laparoscopic approach for removal of worm from biliary canal’.[1] Chalkoo *et al.* succeeded in using a hard laparoscopic procedure for removal of the worm *Ascaris* spp. Chalkoo *et al.* concluded that “with advancement and more familiarity in laparoscopic surgeries, we advocate laparoscopic removal of dead worms in biliary ascariasis in failed ERCP cases.”[1] Indeed, the laparoscopic approach for removal of parasite from biliary tract is mentioned as an effective alternative. Effectiveness of this approach is also confirmed in cases of parasitosis with concomitant biliary stone.[2] However, its use after failure of another non-invasive approach, viz., ERCP, is still questionable. I accept and admire the high surgical skill of Chalkoo *et al.;* however, the rationale of using laparoscopic removal in cases of failed ERCP should be carefully rethought. As Chalkoo *et al.* have noted, the high risk of biliary tract injury during laparoscopic procedure is a big problem preventing success of laparoscopic procedure. In addition, there might be some problems in the selection of proper size of surgical tools for removal of worm from the biliary tract. This might be a repeated problematic area if the surgeon misestimates the size of parasite and the occulted local biliary canal structural disorder. In general, the classical surgical procedure might be more favourable if the surgeon is not highly skilled in laparoscopic practice and does not have good imaging diagnostic tools.
